# Quetiapine-Induced Acute Pancreatitis in a 78-Year-Old Woman

**DOI:** 10.7759/cureus.109390

**Published:** 2026-05-21

**Authors:** Hoyam Elhaj Omer, Ghayda' E'leimat, Urooj Khan, Ashish Valvani

**Affiliations:** 1 Internal Medicine, Michigan State University College of Human Medicine, Flint, USA

**Keywords:** acute pancreatitis, drug-induced acute pancreatitis, metabolic adverse effect, quetiapine, second-generation antipsychotic

## Abstract

Quetiapine-induced pancreatitis is a rare but serious adverse effect of second-generation antipsychotics, often linked to metabolic derangements such as hypertriglyceridemia and diabetic ketoacidosis.

We report a 78-year-old woman found unresponsive at home, with a history of nausea and vomiting in the preceding days. Laboratory evaluation revealed significantly elevated lipase and liver enzymes, and imaging confirmed acute pancreatitis. She had experienced multiple prior episodes of pancreatitis during long-term quetiapine therapy. Common causes, including alcohol use, gallstones, hypertriglyceridemia, hypercalcemia, and autoimmune disease, were rigorously excluded. Medication review revealed long-term quetiapine use. Quetiapine was discontinued during hospitalization, leading to rapid clinical improvement, and no other medications or risk factors were implicated.

The temporal relationship between quetiapine use and symptom onset, along with resolution upon drug withdrawal, suggests probable quetiapine-induced pancreatitis. The recurrence during prolonged therapy highlights the potential for cumulative or direct drug toxicity, independent of metabolic derangements, and underscores the importance of monitoring for adverse effects in high-risk populations.

Clinicians should be aware that quetiapine can rarely cause recurrent acute pancreatitis, possibly via direct toxicity, and maintain vigilance in elderly patients or those on long-term therapy.

## Introduction

Acute pancreatitis is a frequent gastrointestinal emergency with a wide range of causes [1]. The most prevalent etiologies include gallstones, chronic alcohol intake, and hypertriglyceridemia [2]. In a smaller percentage of cases, medications are responsible, with estimates suggesting they account for up to 2% of occurrences [3]. Second-generation antipsychotics (SGAs), such as quetiapine, olanzapine, and risperidone, have well-known metabolic side effects such as hyperlipidemia and hyperglycemia. Although rare, these side effects of SGAs can lead to serious complications, such as hypertriglyceridemia-induced acute pancreatitis, of which there are limited reported cases [4]. Quetiapine has been identified in several pharmacovigilance databases as a potential pancreatotoxic agent, especially in chronic users [1]. Additionally, clinical case reports and retrospective analyses have identified temporal associations between the initiation of SGAs (including quetiapine) and the onset of acute pancreatitis symptoms, suggesting a possible causal relationship [5]. One proposed mechanism involves SGA-induced metabolic abnormalities such as hyperglycemia, insulin resistance, and elevated lipid levels [6]. Similar metabolic alterations have been well described as risk factors for non-drug-induced pancreatitis as well [3]. Quetiapine, an SGA, has been associated with serious metabolic side effects, including hyperglycemia, diabetic ketoacidosis, and, in rare instances, acute pancreatitis, particularly in patients with pre-existing metabolic vulnerabilities [7]. Individuals with pre-existing metabolic syndrome or obesity are particularly susceptible to these secondary effects [6]. Elderly individuals may be particularly vulnerable to the adverse metabolic consequences of SGAs due to age-related reductions in hepatic metabolism and the burden of polypharmacy [8]. Drug-induced pancreatitis (DIP) is typically a diagnosis of exclusion, requiring the careful elimination of other known causes [9]. This is especially critical in patients with no gallstones or alcohol use history but who are on multiple medications [3]. Nitsche et al. emphasized that DIP is often underdiagnosed due to nonspecific symptoms and limited awareness among clinicians [3]. The heterogeneity in clinical presentation further complicates timely diagnosis [9]. Badalov and colleagues developed a classification framework for evaluating DIP causality, categorizing SGAs as a lower but noteworthy risk group [10]. Spanier et al. estimated that nearly 10% of idiopathic pancreatitis cases may actually have a drug-related origin upon comprehensive medication review [11]. Wu and Banks highlighted that early identification and withdrawal of the offending drug can significantly reduce recurrence and improve prognosis [12]. Our case is an example of quetiapine-induced pancreatitis after excluding all other differential diagnoses.

## Case presentation

A 78-year-old woman with a past medical history of abdominal aortic aneurysm, chronic obstructive pulmonary disease, hypertension, and prior cerebrovascular accident was brought to the emergency department after being found down in her bathroom. Her family reported that she had experienced repetitive bouts of nausea and vomiting over the past two days. She also noted similar episodes in the past, which coincided with the use of quetiapine. On presentation, she appeared ill and toxic, in severe respiratory distress, with pallor and cool, mottled skin. Vital signs showed hypertension (blood pressure of 132/103 mmHg), tachycardia (heart rate of 115 beats per minute), and hypoxia with an oxygen saturation in the 60s. She was initially placed on a non-rebreather mask, which was escalated to bilevel positive airway pressure due to persistent hypoxemia. Pulmonary examination revealed rhonchi, while cardiovascular examination showed a regular tachycardic rhythm with normal heart sounds. The abdomen was distended, soft, and tender to palpation with the maximal point of tenderness in the epigastrium. Neurologically, she was alert, oriented, following commands, and moving all extremities equally.

During her emergency evaluation, she developed hypotension concerning for septic shock. She was treated with intravenous fluids, norepinephrine, and broad-spectrum antibiotics, including cefepime and vancomycin, and admitted to the intensive care unit for further monitoring. Laboratory evaluation revealed marked leukocytosis (white blood cell count 32.5×10⁹/L) and thrombocytosis (platelets 455×10⁹/L). Renal function showed acute kidney injury with a creatinine of 2.4 mg/dL, a blood urea nitrogen of 37 mg/dL, and an estimated glomerular filtration rate of 20 mL/min. Metabolic evaluation demonstrated hyperglycemia (glucose 200 mg/dL). Liver function tests were significantly elevated: aspartate aminotransferase 586 U/L, alanine aminotransferase >400 U/L, and alkaline phosphatase 480 U/L. Serum lipase was markedly elevated at 1488 U/L, while triglycerides were 171 mg/dL. Arterial blood gas showed a potential of hydrogen of 7.30, a partial pressure of carbon dioxide of 59 mmHg, and a bicarbonate of 28 mmol/L. Serum lactate was 3.4 mmol/L. The patient's laboratory values are summarized in Table 1. Review of medications showed that the patient was taking quetiapine 100 mg at night for mood disorder.

**Table 1:** The patient's lab level at presentation with the reference range L: liter; mg/dL: milligrams per deciliter; mL/min: milliliters per minute; U/L: units per liter; mmol/L: millimoles per liter; pg/mL: picograms per milliliter; mmHg: millimeters of mercury

Test	Result	Reference range
White blood cell count	32.5×10⁹/L	4.0-10.0×10⁹/L
Platelet count	455×10⁹/L	150-400×10⁹/L
Creatinine	2.4 mg/dL	0.6-1.2 mg/dL
Blood urea nitrogen	37 mg/dL	7-20 mg/dL
Glucose	200 mg/dL	70-110 mg/dL (fasting)
Estimated glomerular filtration rate	20 mL/min	≥60 mL/min
Aspartate aminotransferase (AST)	586 U/L	10-40 U/L
Alanine aminotransferase (ALT)	>400 U/L	7-56 U/L
Alkaline phosphatase (ALP)	480 U/L	40-129 U/L
Lipase	1488 U/L	13-60 U/L
Lactate	3.4 mmol/L	0.5-2.2 mmol/L
N-terminal prohormone of brain natriuretic peptide (NT-proBNP)	500 pg/mL	<125 pg/mL (age <75 years)
Arterial blood gas: pH	7.30	7.35-7.45
Arterial blood gas: partial pressure of carbon dioxide (pCO₂)	59 mmHg	35-45 mmHg
Arterial blood gas: bicarbonate (HCO₃⁻)	28 mmol/L	22-26 mmol/L
High-density lipoprotein cholesterol (HDL-C)	22 mg/dL	≥40 mg/dL
Triglycerides	171 mg/dL	<150 mg/dL
Low-density lipoprotein cholesterol (LDL-C)	32 mg/dL	<100 mg/dL
Total cholesterol	88 mg/dL	<200 mg/dL

Imaging studies included chest X-ray, which revealed bilateral lower lobe and right upper lobe infiltrates, and computed tomography (CT) of the abdomen and pelvis, which demonstrated mural thickening of the duodenum, dilation of the biliary and pancreatic ducts, and a stable 1.4 cm cystic lesion in the pancreatic tail. Of note, the abdominal CT did not demonstrate any radiographic evidence of an infected cyst, including no surrounding inflammatory changes, rim enhancement, adjacent fat stranding, or fluid collection (Figures 1-2).

**Figure 1:**
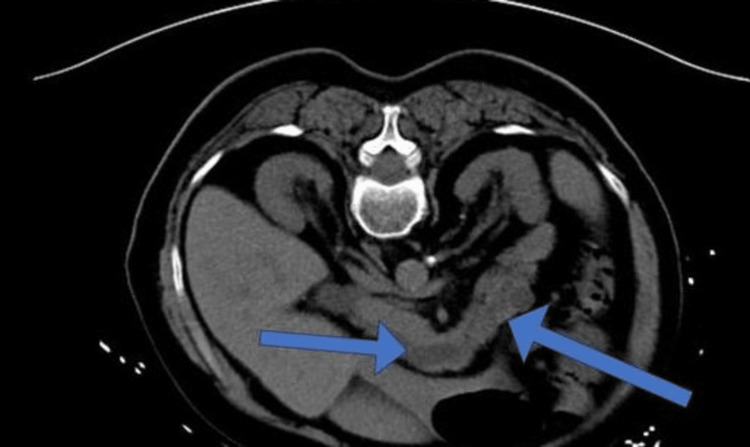
Computed tomography scan of the abdomen without contrast showing the dilation of the biliary and pancreatic ducts and a 1.4 cm cystic lesion in the pancreatic tail

**Figure 2:**
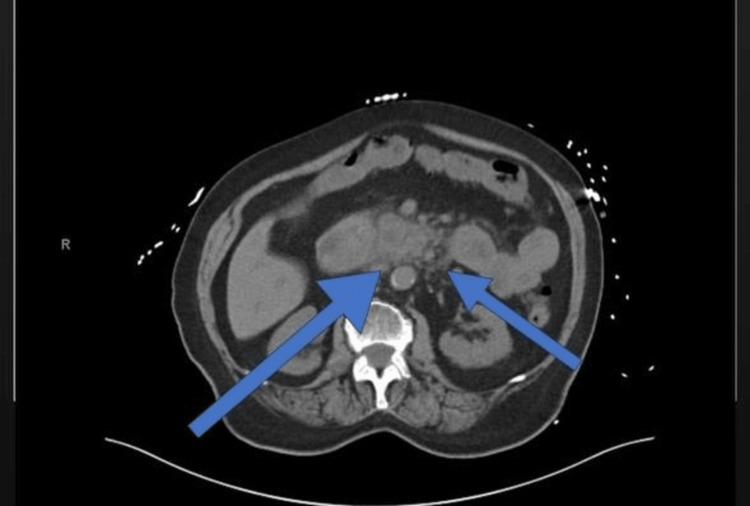
Computed tomography scan showing the dilation of the biliary and pancreatic ducts and the cystic lesion in the pancreatic tail

Review of prior medical records revealed two previous episodes of acute pancreatitis, occurring in 2014 and 2024, both temporally associated with quetiapine use, with the latter episode occurring approximately one year prior to the current presentation. Although the exact duration of quetiapine use prior to each pancreatitis episode could not be determined from the available records, the patient reported that quetiapine had been initiated before the onset of both episodes. Importantly, quetiapine therapy was not discontinued following either prior episode, as no definitive alternative etiology was identified at the time and the medication-related association had not yet been recognized. During the 2024 episode, she presented with nausea, vomiting, left upper quadrant pain, and markedly elevated lipase (>3,000 units/L). Abdominal ultrasound demonstrated ductal dilation, while magnetic resonance cholangiopancreatography revealed diffuse pancreatic ductal dilation with small cysts in the pancreatic tail, without choledocholithiasis. Esophagogastroduodenoscopy was performed as part of the evaluation for persistent gastrointestinal symptoms and demonstrated hypertrophic, erythematous duodenal mucosa, with biopsies showing ulceration and acute inflammation. Autoimmune workup, including immunoglobulin G subclass 4 testing, was negative. Lipid profile was notable only for low high-density lipoprotein, and the hepatitis panel was negative. Despite extensive evaluation during both prior episodes, no alternative etiology was identified, further supporting quetiapine as the probable cause of recurrent pancreatitis. During the current admission, the patient was treated for community-acquired pneumonia and acute pancreatitis with supportive care, including oxygen therapy, bilevel positive airway pressure, intravenous fluids, and pain control, in addition to intensive care unit monitoring for multi-organ involvement. Quetiapine was discontinued given its potential association with recurrent pancreatitis. Over the course of her intensive care unit stay, the patient stabilized, with gradual improvement in renal function and pancreatic enzyme levels. This case highlights recurrent acute pancreatitis in an elderly patient potentially induced by quetiapine, particularly in the setting of prior structural pancreatic abnormalities and multi-organ involvement, emphasizing the importance of reviewing medications in patients with recurrent pancreatitis episodes.

## Discussion

DIP is an uncommon but recognized form of acute pancreatitis that should be considered once common etiologies, such as gallstones, alcohol abuse, and hypertriglyceridemia, have been excluded [1]. Although most cases of acute pancreatitis are attributable to biliary disease or alcohol, DIP accounts for approximately 0.1-2% of cases, making it an important differential diagnosis in idiopathic presentations [2]. Despite this, DIP is frequently overlooked due to the absence of specific clinical markers and the need for diagnosis by exclusion [3].

Quetiapine, an SGA, has been implicated in individual case reports of acute pancreatitis, with symptoms typically resolving following the discontinuation of the medication. Recent pharmacovigilance data from large-scale national databases have further supported this association, identifying quetiapine as a potential pancreatotoxic agent, particularly among chronic users. For instance, Noguchi et al., using the Japanese claims database, reported significant associations between multi-acting receptor-targeted antipsychotics, including quetiapine, and the occurrence of acute pancreatitis in real-world settings [5]. Additionally, it has been illustrated that even low-dose quetiapine may precipitate acute pancreatitis in susceptible individuals.

The mechanism underlying quetiapine-associated pancreatitis is not fully understood; however, SGAs are known to cause significant metabolic effects, including hyperglycemia, insulin resistance, and dyslipidemia, which may indirectly contribute to pancreatic inflammation [6]. SGAs such as quetiapine can therefore trigger metabolic dysregulation and, in rare cases, pancreatitis, particularly in patients with pre-existing metabolic vulnerabilities [7]. Elderly patients may be especially susceptible to DIP due to age-related decline in drug metabolism and increased comorbidities [8].

Given these diagnostic challenges, a comprehensive medication history is essential in all cases of idiopathic pancreatitis, especially when standard etiologies are absent [9]. This includes detailed history-taking and metabolic assessment in patients with unexplained pancreatitis [2]. Accordingly, clinicians should maintain a high index of suspicion for DIP, particularly in polymedicated or elderly patients [3]. To aid in evaluating drug causality, a structured classification system has been proposed that categorizes drugs based on the quality of available evidence; SGAs fall under class II or III depending on recurrence and case quality [10]. In a large retrospective analysis, it was estimated that approximately 10% of idiopathic pancreatitis cases could be linked to medication use upon thorough review [11]. Importantly, excluding a clear etiology can delay appropriate treatment and increase the likelihood of recurrence [1], whereas timely discontinuation of the offending drug significantly reduces the risk of complications, intensive care unit admission, and recurrence [12].

Quetiapine-induced pancreatitis has previously been associated with metabolic derangements, including hypertriglyceridemia and diabetic ketoacidosis [4,7]. We present a unique case of recurrent quetiapine-associated pancreatitis occurring in the absence of significant metabolic abnormalities, suggesting a possible alternative mechanism such as idiosyncratic or direct drug-related pancreatic toxicity. Reports of pancreatitis independent of metabolic derangements remain considerably rarer, and the underlying mechanism is not clearly established in the literature. Additionally, this case raises the possibility that elderly patients may be particularly susceptible to such adverse effects, although further investigation is required.

## Conclusions

This case reinforces the importance of recognizing quetiapine as a rare etiology of acute pancreatitis, especially in elderly patients without traditional risk factors. Clinicians should maintain vigilance for abdominal symptoms in patients on SGAs, ensure routine metabolic monitoring (lipids, glucose, liver enzymes), and discontinue the agent if pancreatitis is suspected.
